# Blood Pressure and Heart Rate Measurements Using Fiber Bragg Grating Sensor with Optical Power Detection Scheme

**DOI:** 10.3390/s25072007

**Published:** 2025-03-23

**Authors:** Yu-Jie Wang, Likarn Wang

**Affiliations:** Institute of Photonics Technologies, National Tsing Hua University, Hsinchu 300, Taiwan; melody2star@gmail.com

**Keywords:** blood pressure, fiber Bragg grating sensor, pulse transit time, pulse ascending time, pulse descending time, optical power detection scheme

## Abstract

A low-cost dual-FBG (fiber Bragg grating) architecture is employed to capture the pulse waveform of the artery at the subject’s wrist by measuring changes in optical power. The pulse transit time (PTT), pulse ascending time, and pulse descending time extracted from the pulse waveform are used in a blood pressure (BP) estimation model by fitting the measured BP with the reference BP obtained from a commercial sphygmomanometer. The estimation model is developed using data from 29 subjects at the age of 20 to 54. The results demonstrate that the errors between the calculated values and reference values of SBP and DBP for all of the 29 subjects both range from −4 to 5 mmHg with mean errors of 0.72 mmHg and 0.83 mmHg, respectively. The standard error can be found to be 2.45 and 2.59 mmHg for SBP and DBP, respectively. Also, it is found that this BP estimation model outperforms two BP models derived by considering PTT only.

## 1. Introduction

There are two methods for measuring blood pressure in human bodies: invasive and non-invasive methods. Sometimes, patients in intensive care units or operating rooms would need arterial cannulation (AC) to continuously monitor their blood pressure, nevertheless at the risk of getting infected [1]. On the other hand, non-invasive methods of blood pressure measurement have been extensively studied. They can be categorized into continuous and non-continuous types. As we know, a non-invasive commercial blood pressure measuring device extensively adopted by the public is based on the use of the oscillometric measurement with a cuff inflated and deflated around the subject’s arm [2,3]. This method is not continuous and may cause inconvenience to particular patients. Many continuous blood pressure measurement methods have been studied. The photoplethysmography-based (PPG-based) device using light sources and photodiodes to detect arterial pulse waves equipped on wrists was tested on patients immediately following cardiac surgery, demonstrating maximum discrepancies of −3.3 beat/min in heart rate and 6–10 mmHg in systolic blood pressure, compared with AC measurements [4]. PPG has been used for blood pressure measurement for several decades [5] and recently gained popularity for wearable devices; however, it is usually used only in non-movement situations [6,7]. Blood pressure could also be measured by PPG with the help of the electrocardiograph (ECG) method to derive pulse transit time (PTT) that relates to blood pressure [8,9].

On the other hand, fiber optic solutions for the acquisition of arterial pulse waveforms have been adopted to assess the health of subjects. Fiber Bragg gratings (FBGs) were mostly used for the purpose, such as in the work of [10,11], which measured, respectively, the pulse waveform of carotid arteries and the waveforms of carotid and foot dorsal arteries to acquire the PTT between the carotid and foot. Also, blood pressure and heart rate were measured non-invasively by using FBG sensors by other researchers [12,13,14]. These studies measured vascular pulse waveforms by detecting changes in the reflection wavelength of the FBG in order to obtain the values of blood pressure and heart rates. Additionally, in 2019, an optical method combined with a blood pressure calibration model for measuring blood pressure was provided. There, a single fiber grating was used to sense vascular pulsation signals, and a Mach–Zehnder interferometer was employed to obtain the pulsation signals of pulse waveforms. These waveforms were then processed by a blood pressure value calculation unit to determine blood pressure values [15]. A wearable device with two sets of fiber gratings being placed on the wrist was used to measure the blood pressure. This device detected changes in wavelength and estimated blood pressure values in cooperation with a blood pressure estimation model [16].

The works mentioned above for the acquisition of vascular pulse waveforms by using FBGs are just a few. All of them, except that of [15], used a spectrometer to obtain the time-domain variation of the reflection wavelength (i.e., the Bragg wavelength) of the FBG in order to obtain the pulse waveforms. These detection methods used a broadband light source and acquired the shift of Bragg wavelength resulting from the vascular pulsation-induced strain by using a spectrometer. When it comes to a strain measurement, an optical power detection scheme in a proper design can calibrate the strain, as mentioned in the work of [17,18]. In the system of [17], two FBGs were cascaded, wherein the optical power corresponding to the union of the reflection spectra of the two FBGs was used to calibrate the strain imposed on one FBG. In the power detection scheme in the work of [18], the overlapped part between the reflection spectra of the two FBGs corresponded to the strain to be measured. However, the method used in [18] would lead to a large optical loss due to the use of double 3 dB couplers for acquiring the overlapped part between the reflection spectra of the two FBGs.

Here, in this research, we use two identical FBGs to calibrate the vascular pulsation-induced strain and accordingly acquire the pulse waveform based on a different optical power detection scheme. However, the overlapped part between the reflection spectrum of one FBG (the sensing FBG) and the transmission spectrum of the other FBG is used to obtain the optical power that depends on the vascular pulsation-induced strain. There is a difference between this optical power detection scheme and that used in the method of [17]. When the two FBGs suffer from no vascular strain, the optical power detected would approach null by using this new detection scheme, whereas it would not with the method of [17]. Therefore, the new detection scheme offers a more sensitive strain-sensing capability in detecting the vascular pulse.

In Section 2, we introduce the principle of the proposed optical method for measuring vascular pulsation waveform. Blood pressure and heart rate measurements based on the dual FBG method are shown in Section 3, and then blood pressure measurement data are demonstrated in Section 4. In Section 4, two different blood pressure models are used to give different results. Section 5 concludes this work.

## 2. Outline of the Presented Measurement System

### 2.1. Pulse Transit Time (PTT)

It was stated by Peter et al. that blood pressure was related to the PTT, a time delay between the R-wave peak of ECG and the first peak of the pulse waveform acquired by PPG on the finger [19]. Simultaneous acquisition of ECG and PPG signals may seem to be complicated and inconvenient for subjects. Later, it was verified that the pulse waveform obtained from the wrist artery of a subject can determine the blood pressure of the subject by using the PTT defined as the time delay between the first and second peaks of the pulse waveform, which are, respectively, the peaks of the percussion wave and the dicrotic wave [20]. The systolic pressures and diastolic pressures of seven subjects were measured and compared with those obtained by an OMRON commercial instrument (purchased from Japan). Although the results for the seven subjects by using the method of [20] conformed to the required accuracy by The Association for the Advancement of Medical Instrumentation (AAMI), the accuracy of those predicted systolic pressures was within ±7 mmHg, indicating that more accurate pressure levels are still required.

Here, below, we present an FBG-based optical power detection scheme to calibrate the vascular pulsation-induced strain, wherein PTT is determined as in [20] by using the acquired pulse waveform. The FBG sensor is applied to the radial arteries of 29 subjects, and blood pressures, including systolic and diastolic blood pressures, as well as heart rates, are calculated as a function of PTT and other parameters acquired from the pulse waveform.

### 2.2. Optical Power Detection for Blood Pressure Measurement

**Figure 1:**
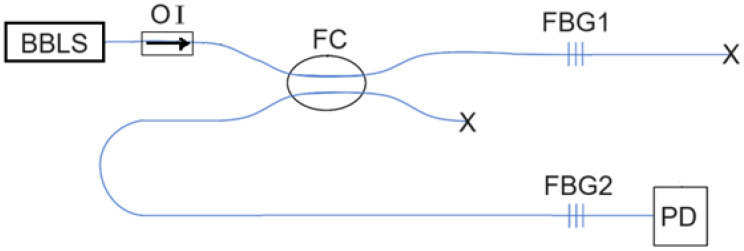
Optical power detection scheme to acquire vascular pulsation waveform with two identical FBGs used. BBLS: broadband light source; OI: optical isolator; FC: 3 dB fiber coupler; FBG1 and FBG2: fiber Bragg gratings; PD: photodetector. The symbol X denotes no Fresnel reflection from fiber end faces.

Figure 1 shows our presented optical power detection scheme based on the use of two FBGs with the same Bragg wavelength at 1546.92 nm and the same reflection bandwidth of 1 nm. Both FBGs have a reflectivity of 90%. FBG 1 senses the vascular pulsation-induced strain, while FBG2 does not. A broadband light source (BBLS), which is a 30 m long erbium-doped fiber pumped by a 980 nm laser of 70 mW through a WDM coupler, provides a flat enough ASE spectrum from 1545 nm to 1549 nm. FBG1 receives a broadband light through a fiber coupler (FC) and reflects a part of the light at its reflection band that then returns to the FC and is directed to FBG2. An optical isolator (OI) is used to avoid any light reflected back into the BBLS. Additionally, two fiber end faces marked by a symbol X denote that a part of fiber near the end face is bent to have no Fresnel reflection from each end face.

**Figure 2:**
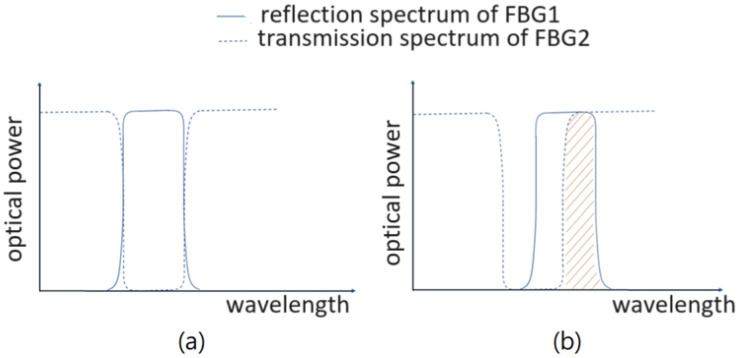
Reflection spectrum of FBG1 and the transmission spectrum of FBG2 (**a**) when FBG1 does not bear any vascular pulsating stress, and (**b**) when FBG1 bears some vascular pulsating stress.

A photodetector (PD) receives the light transmitted through FBG2 and generates an electrical signal of a pulse waveform of vascular pulsation in response to the stress incurred on FBG1 due to vascular pulsation. The wavelength band shifts with the vascular pulsating stress at the wrist of a subject to be measured. The two FBGs operate as follows. Figure 2a is a schematic diagram showing the reflection spectrum of FBG1 (RB1) and the transmission spectrum of FBG2 (TB2) when FBG1 is not affected by vascular pulsating stress. For the case that FBG1 and FBG2 have the same reflection wavelength band, RB1 and TB2 are complementary. If FBG1 does not bear any vascular pulsating stress, the wavelength band of the light reflected from FBG1 is almost completely within the reflection band of FBG2. In other words, the optical power of the light received by the PD is very small or almost zero. When FBG1 bears some vascular pulsating stress such that RB1 shifts to the right, RB1 will partially overlap the high-power part of TB2, as shown in Figure 2b. The overlapped part of RB1 and TB2 is indicated by the slash region, the area of which is directly proportional to the optical power of the light passing through FBG2 and detected by the PD.

If FBG1 bears a lower vascular pulsating stress, the area of the slash region is smaller, the optical power of the light passing through FBG2 is then lower, and, thus, the electrical signal outputted by the PD would be smaller. On the contrary, if the FBG1 bears a larger vascular pulsating stress, the area of the slash region becomes larger, and then the optical power of the light passing through FBG2 becomes higher, causing the electrical signal outputted by the PD to become larger. That is to say, when the vascular pulsating stress applied to FBG1 fluctuates, the electrical signal outputted by the PD fluctuates, thereby forming the pulse waveform of vascular pulsation.

### 2.3. Fiber Bragg Grating Sensor Embedded in Silicone Sheet

The optical circuit shown in Figure 1 for blood pressure and heart rate measurement contains two FBGs, with one being strained by vascular pulsation stress and shifting its reflection wavelength band, thereby changing the optical power detected by the photodetector to obtain a pulse waveform signal. This FBG (i.e., FBG1 in Figure 1, also referred to as FBG sensor herein), which serves as the pulsation sensor of the blood vessel at the wrist, must be close to the blood vessel. However, in order to prevent the FBG sensor and its adjacent bare fiber from being damaged or broken when placing and moving the FBG sensor close to the radial artery of the wrist, the bare fiber containing the FBG is fusion spliced to a loose-tube fiber, and then the bare fiber and a small part of the loose-tube fiber are embedded in a silicone sheet. When blood pressure and heart rate are measured, the silicone is put in the right place, and the FBG sensor is close to the skin next to the radial artery of the wrist to obtain the pulse waveform signal.

**Figure 3:**
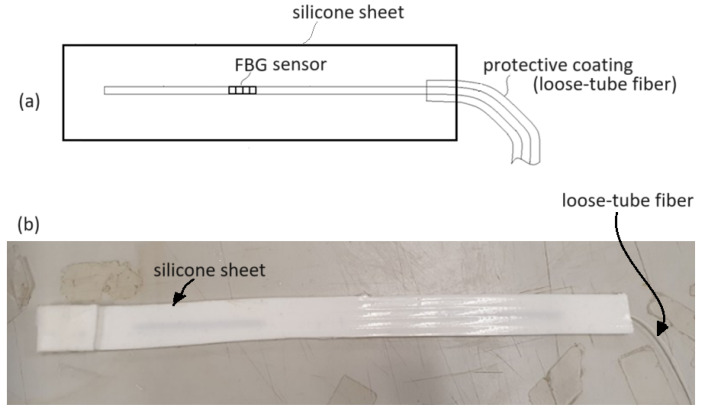
(**a**) Schematic diagram showing a protective structure for protecting an FBG sensor and its adjacent bare fiber. (**b**) The silicone sheet with an FBG, its adjacent bare fiber, and a part of loose-tube fiber embedded inside.

Figure 3a is a schematic diagram showing a protective structure for protecting the FBG sensor and its adjacent bare fiber, which are embedded in a silicone sheet with a thickness of 3 mm. To protect the fiber outside the silicone sheet, a loose-tube fiber is spliced to the bare fiber inside the silicone sheet with a small part of it embedded inside the silicone sheet as well. The loose-tube fiber is a single-mode fiber with a protective coating such that the fiber is less susceptible to breaking. To reduce the Fresnel reflection from the end face of the bare fiber, a part of the fiber near the end face has a bending loss of light by presenting a curled state with a small radius of curvature. Figure 3b shows the silicone sheet within which an FBG sensor, its adjacent bare fiber, and a part of loose-tube fiber are embedded. The light can be emitted to the FBG sensor from the loose-tube fiber outside the silicone sheet through a bare fiber inside the silicone sheet. Then, the light at the reflection wavelength band of the FBG sensor (i.e., FBG1 in Figure 1) is reflected back to the loose-tube fiber outside the silicone sheet. The light is finally transmitted to the photodetector to obtain the pulse waveform signal when the FBG sensor bears a vascular pulsation stress.

## 3. Measurement of Blood Pressure and Heart Rate

**Figure 4:**
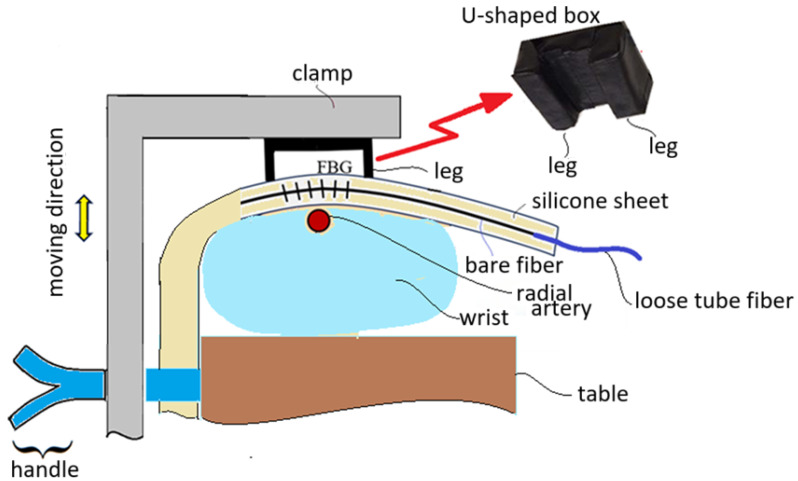
Schematic diagram showing a silicone sheet containing an FBG sensor is pressed by a U-shaped box with a clamp to closely contact the skin of wrist.

When the FBG sensor embedded inside a silicone sheet is positioned near the radial artery of a subject’s wrist, a U-shaped box is used to press the silicone sheet to not only make the silicone sheet closely contact with the skin of the wrist but also to enhance the vascular pulsation sensing capability. The U-shaped box is pressed by a clamp to make its legs tightly touch the silicone sheet, as shown in Figure 4. This figure schematically shows that the clamp can be moved up and down by controlling a handle. The wrist is put on a table and the handle touches firmly the side of the table for the clamp to move up and down. When the clamp is moved downwards to press the U-shaped box, the legs of the U-shaped box tightly touch the silicone sheet. When the clamp is moved upwards, the wrist of the subject can move out.

**Figure 5:**
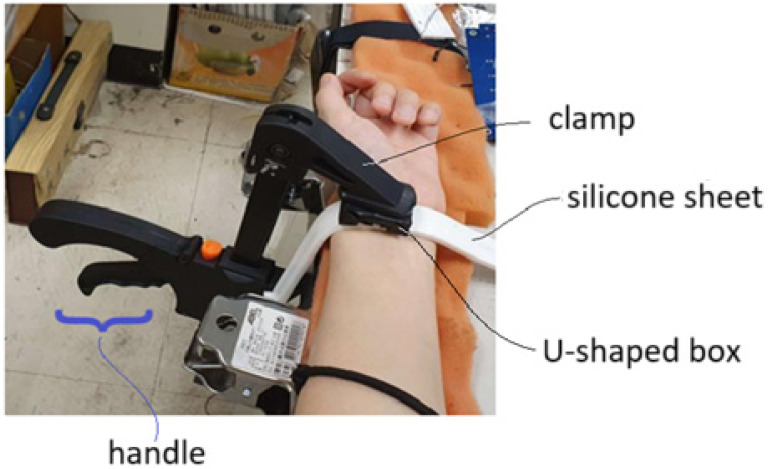
An arm of a subject is fixed by a clamp in acquiring vascular pulsation waveform.

The arm of a subject is kept stable with the clamp shown in Figure 4 in the measurement of blood pressure and heart rate, as shown in Figure 5, where the handle is held and pressed to keep the arm clamped and the U-shaped box tightly touching the silicone sheet. The FBG sensor across the legs of the U-shaped box can then sense the vascular pulsation stress, and pulse waveforms are obtained at the output of the PD with the clamping scheme.

The pulse waveforms acquired by the PD are inputted to an analog-to-digital converter, and then the microcontroller ESP32 is used to sample the pulse waveform at a sample rate of 200 Hz. The sampled signals are then transferred to a computer instantaneously.

**Figure 6:**
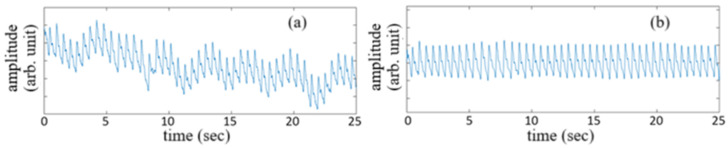
Vascular pulsation waveform of a subject acquired for a time period of 25 sec before signal treatment (**a**) and after signal treatment (**b**).

The vascular pulsation waveforms of a subject acquired for 25 sec before signal treatment and after signal treatment are shown by the traces in Figure 6a,b, respectively. The untreated signal waveform corresponds to the detected originally by the PD, while the use of a bandpass filter with a band from 0.5 Hz to 12 Hz for the untreated signal waveform gives the vascular pulsation waveform shown in Figure 6b.

**Figure 7:**
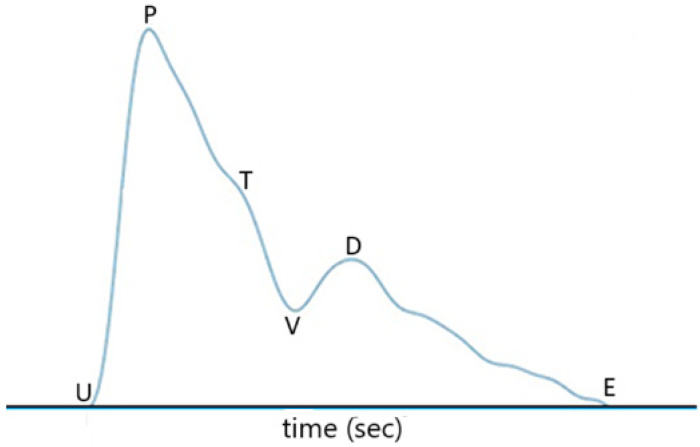
A pulse waveform detected by the PD. U point is the onset point for blood ejection from left ventricle when the heart contracts, P is the peak of the percussion wave during systole, T represents the tidal wave, V is the valley of the pulse representing the beginning of diastole (i.e., the **descending wave trough point**), D is the peak of the dicrotic wave, and point E represents the end of diastole.

Each pulse of the filtered signal waveform shown in Figure 6b can be used for the estimation of blood pressure and heart rate. All of the pulses acquired during a certain time period, which is set to be 25 sec in this study, almost have the same shape as that shown in Figure 7, where U represents the onset point for blood ejection from the left ventricle when the heart contracts, P is the peak of the percussion wave during systole, T represents the tidal wave, V is the valley of the pulse representing the beginning of diastole (i.e., the **descending wave trough point**), D is the peak of the dicrotic wave, and point E represents the end of diastole. In Figure 7**, the time interval between point P and point D is defined as PTT, the same as in** [20]**. The time interval from point U to point P is defined as Pt, named by pulse ascending time herein. The time interval from point D to the end point E of the pulse waveform, which is normally the onset point of the next pulse waveform, is defined as Dt, named by pulse descending time herein. The heart rate can be obtained from the repetition rate of the filtered pulse waveform signal in** Figure 6**. In other words, the heart rate can be obtained from the repetition rate of point U, point P, point V, or point D of the pulse waveform.**

The difference between SBP and DBP is related to PTT, the density of blood, and the volume of blood in the artery, as indicated by Equation (2) of Ref. [20]. From some experimental results, mean BP and PTT are related according to Equation (6) of Ref. [20]. SBP and DBP are related to PTT in accordance with Equations (6)–(8) of Ref. [20]. On the other hand, we believe that the pulse ascending time would be related to SBP because this time parameter has something to do with the time required for blood ejection from the heart. Likewise, the pulse descending time is related to the time for diastole. Therefore, in the present study, we add Pt and Dt in the BP estimation model.

Because the processed pulse waveform signal has multiple percussion wave peaks within a time period and the three time parameters PTT, Pt, and Dt provided by the pulse waveforms of these different peaks will be slightly different, it is necessary to use an averaging method to obtain these time parameters. The averaging method here is to take out the time parameter values of each pulse waveform and then average the time parameters of these pulse waveforms to give a set of definite time parameter values. Systolic blood pressure (SBP) and diastolic blood pressure (DBP) can then be calculated using the three parameters PTT, Pt, and Dt of the pulse waveform signal. Equations (1) and (2) are those we use for the calculation of systolic and diastolic blood pressures, wherein *ln* is a natural logarithmic function, the values of the coefficients A, B, C, D, E, F, G, and H are obtained by using a regression method of

(1) $$SBP=A\times ln\left(PTT\right)+C\times \frac{1}{{PTT}^{2}}+E\times Pt+G$$

(2) $$DBP=B\times ln\left(PTT\right)+D\times \frac{1}{{PTT}^{2}}+F\times Dt+H$$

nonlinear least squares. In this method, vascular pulse waveforms of 29 subjects are obtained using the proposed dual-FBG power detection scheme to determine their **three parameters—PTT, Pt, and Dt—and their** blood pressures (referred to as reference pressures) are **measured by the commercial blood pressure monitor OMRON model** HEM-FL31 (which can also measure heart rate). **Then, the unknown coefficients A, B, C, D, E, F, G, and H of Equations (1) and (2) are determined to give the minimum discrepancy between the calculated blood pressure values (using the two equations) and the reference pressure values over all of the 29 subjects**.

## 4. Blood Pressure Measurement Data

### 4.1. Using Pulse Transit Time Only for Blood Pressure Determination

We first follow the postulation of the researchers of Ref. [21] in determining blood pressure by using only PTT. That is, we set E = F = 0 in Equations (1) and (2). Using nonlinear least-square regression, we find the best set of the coefficients A = 680.21, B = 801.52, C = 25.77, D = 28.28, G = 648.44, and H = 731.92 for 29 subjects whose PTT values derived from their pulse waveforms are shown in Table 1. In the table, we also show values of Pt and Dt of their pulse waveforms for later use. But, here, the heart rates of these subjects derived from pulse waveforms and those obtained by OMRON are also shown for comparison.

The subjects under test are six females and 23 males, with 24 of them aged between 20 and 23, three of them being 27 years old, and two of them above 50. It should be noted that all of the 29 subjects are tested to give their values of PTT, Pt, Dt, and BP. Nevertheless, only the heart rates of the first 21 subjects are recorded here because of our incautiously missing data. From Table 1, we can see that the heart rates of the first 21 subjects are determined by the pulse waveforms with an error within ±5 s^−1^, with respect to those obtained by OMRON.

**Figure 8:**
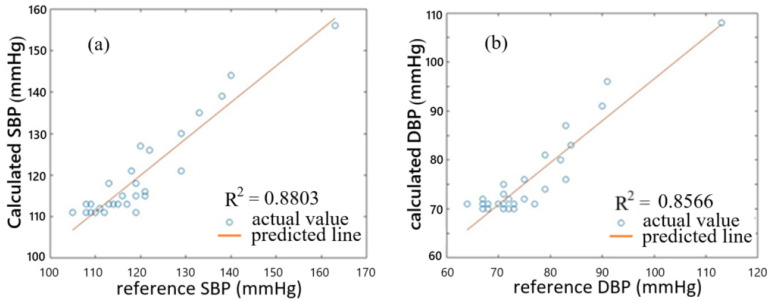
Correlation plots of calculated BP values against reference BP values for 29 subjects. (**a**) is for SBP and (**b**) for DBP.

**Figure 9:**
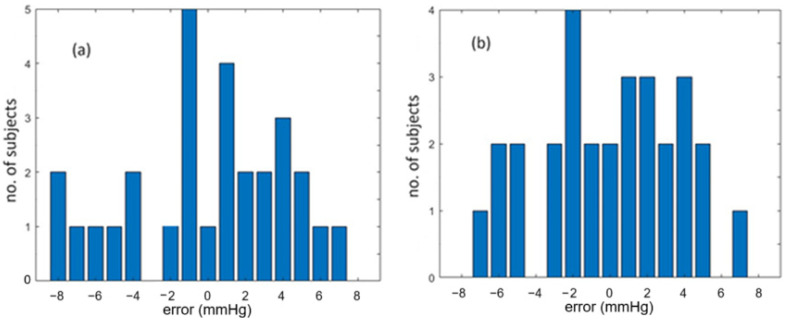
Error distributions of calculated BP values and reference BP values for the 29 subjects. (**a**) is for SBP and (**b**) for DBP.

By using the BP estimation model with only PTT used, we obtained the coefficients required by Equations (1) and (2), as stated previously. The calculated SBP and DBP are then estimated using the equations. Figure 8a,b shows the correlation plots of calculated BP values against reference BP values for the 29 subjects. The correlation coefficients of SBP and DBP are 0.8803 and 0.8566, respectively, indicating that the calculated BP values and reference BP values are close to each other in some sense for the 29 subjects. Figure 9 shows the error distribution of calculated BP values and reference BP values. From this figure, we can see that the errors of SBP range from −8 to 7 mmHg, while the errors of DBP are from −7 to 7 mmHg. The mean error and standard error of SBP are 0 mmHg and 4.12 mmHg, respectively, while the mean error and standard error of DBP are −0.03 mmHg and 3.65 mmHg, respectively. Although the average accuracy achieved by the proposed fiber-optic method here fulfills the required accuracy of ±5 mmHg by AAMI, we can find that several subjects, such as subjects F, K, R, U, X, and Z3, have an over-estimated BP, which has an error larger than ±5 mmHg. Therefore, we need a better BP estimation model.

Since the blood pressure would be higher if blood ejection goes faster during heart contraction, we include a parameter describing the time period for vascular pulsation to reach the peak from the onset point. This time period is defined previously as pulse ascending time, denoted by Pt. Also, pulse descending time Dt plays a role in determining blood pressure. Therefore, we consider these parameters in addition to PTT in BP modeling in Section 4.2.

### 4.2. Considering Pt and Dt as Well to Obtain BPs

**Figure 10:**
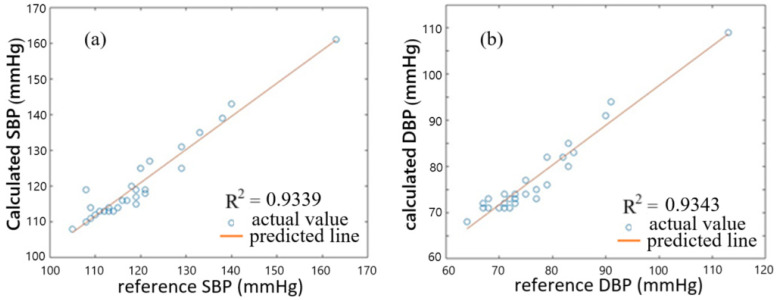
Correlation plots of calculated BP values against reference BP values for 29 subjects. (**a**) is for SBP and (**b**) for DBP.

**Figure 11:**
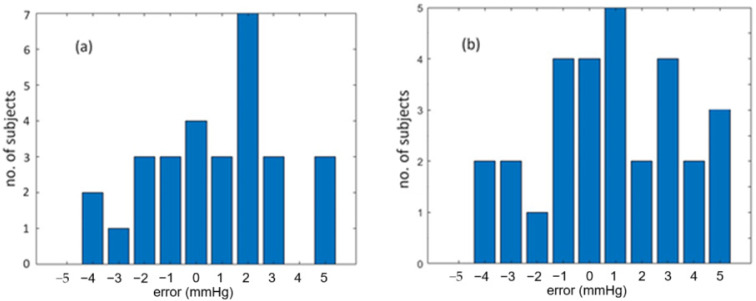
Error distributions of calculated BP values and reference BP values for the 29 subjects. (**a**) is for SBP and (**b**) for DBP.

To use a better BP estimation model, we consider PTT, Pt, and Dt of the 29 subjects, which are shown in Table 1, in deriving a new set of coefficients A, B, C, D, E, F, G, and H in Equations (1) and (2). Again, using the method of nonlinear least-square regression, we obtain A = 671.22, B = 814.3, C = 25.47, D = 28.69, E = 24.61, F = 8.44, G = 639.2, and H = 741.17. Figure 10a,b show the correlation plots of calculated BP values against reference BP values for the 29 subjects. The correlation coefficients of SBP and DBP are 0.9339 and 0.9343, respectively, indicating that the calculated BP values and reference BP values are closer to each other for the 29 subjects, with respect to the case when only PTT is used for modeling. Error distributions of calculated BP values and reference BP values for the 29 subjects are shown in Figure 11, where (a) is for SBP and (b) is for DBP. It shows that the errors between the calculated values and reference values of SBP and DBP for all of the 29 subjects both range from −4 to 5 mmHg, with mean errors of 0.72 mmHg and 0.83 mmHg, respectively. The standard error can be found to be 2.45 and 2.59 mmHg for SBP and DBP, respectively. The average accuracy achieved by using the present equations fulfills the required accuracy of ±5 mmHg by AAMI for all of the subjects. Compared with the previous estimation model with PTT used only, the present model with PTT, Pt, and Dt used gives more accurate BP estimation. It is noted that the accuracy of SBP is about the same as that using a cuffless measurement based on PTT obtained by PPG and ECG [9]. The work of Ref. [9] reported the mean and standard deviation of −0.34 (or 0.52) mmHg and 3.1 (or 3.3) mmHg for SBP monitoring, in contrast to our results of 0.72 ± 2.45 mmHg.

## 5. Conclusions and Discussion

We have presented a fiber-optic power-detection scheme for blood pressure and heart rate measurement, wherein dual FBGs with one embedded in a silicone sheet for protection are used to sense vascular pulsation. Pulse waveforms in accordance with vascular pulsation are obtained by detecting the optical power proportional to the overlapped area of the reflection spectrum of the sensing FBG and the transmission spectrum of the other FBG.

Two models for blood pressure estimation are used here: first, with pulse transit time considered only, and second, with the consideration of pulse transit time (PTT), pulse ascending time (Pt), and pulse descending time (Dt) in modeling the blood pressure equations. In the estimation of blood pressures of twenty-nine subjects, the first model provides mean errors of 0 mmHg (for SBP) and −0.03 mmHg (for DBP), while the second model provides mean errors of 0.72 mmHg (for SBP) and 0.83 mmHg (for DBP). The calculated values and reference values of SBP (DBP) are related with a correlation coefficient of 0.8803 (0.8566) for the first model with only pulse transit time used for modeling. However, the second model with two additional pulse parameters considered provides the correlation coefficients of SBP and DBP of 0.9339 and 0.9343, respectively, indicating that the calculated BP values and reference BP values are closer to each other for the 29 subjects, with respect to the case when only PTT is used for modeling.

The area of the slash region (spectrally overlapping region) would not change with the ambient temperature because the two FBGs were placed near each other, and, accordingly, their Bragg wavelengths shifted the same amount as the temperature varied. We have carried out an experiment of blood pressure measurement on a subject to see the effect of ambient temperature with the same measurement setup as the present work except that the silicone sheet was shorter and the U-shaped box was pressed by a strap wrapped around the wrist instead of a clamp. At room temperature (24 °C), the three temporal parameters were 0.256 s (PTT), 0.1325 s (Pt), and 0.189 s (Dt), compared with 0.259 s (PTT), 0.1304 s (Pt), and 0.193 s (Dt) obtained at 45 °C. SBP and DBP were determined by a different estimation model to be 110.3 mmHg and 79.0 mmHg at room temperature and 109.2 mmHg and 77.7 mmHg at 45 °C, in contrast to the reference values of 110 mmHg (SBP) and 78 mmHg (DBP) obtained by an OMRON commercial instrument at room temperature. It can be seen that the measurement setup was stable against temperature variation.

**Figure 12:**
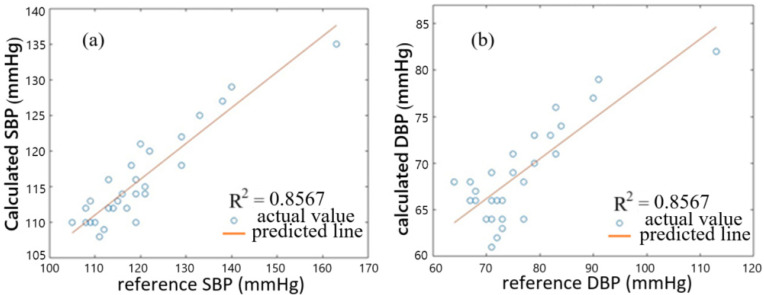
Correlation plots of calculated BP values against reference BP values for 29 subjects. (**a**) is for SBP and (**b**) for DBP.

The work of [21] also used only pulse transit time to model BP equations and estimated SBP and DBP both to within ±4 mmHg for 17 subjects. We have used their model to calculate the SBP and DBP of our 29 subjects using the PTT values listed in Table 1. The calculated values of SBP and DBP for the 29 subjects relate to the reference values of their SBP and DBP not so well as those calculated values for the case when PTT, Pt, and Dt are considered for modeling. Figure 12 shows the correlation plots of calculated BP values against reference BP values. The correlation coefficients are 0.8567 for SBP and 0.6681 for DBP. Also, it can be seen that the errors between calculated SBP (DBP) and reference SBP (DBP) for 6 (13) subjects reach above 8 mmHg. In particular, the errors reach above 11 mmHg for four subjects in the estimation of SBP and reach above 12 mmHg for five subjects in the estimation of DBP.

In [21], the SBP and DBP followed the same forms in Equations (1) and (2) with E = F = 0; however, with different coefficient values of A, B, C, D, G, and H from those values for the first case in the presented study. Specifically, coefficients were A = 189.7, B = 23.8, C = 8.7, D = 3.0, G = 239.6, and H = 54.4 in their study. When the PTT values of the 29 subjects are put into their equations, larger errors are found compared with the results of [21]. This situation might result from the fact that the BP model in [21] was established for those 17 subjects who were healthy as BP is concerned (except one having 135 mmHg SBP). This established BP model might cause slight failure in predicting the BP for a group of subjects having larger BP variations.

When PTT, Pt, and Dt are used for estimation, errors with 5 mmHg are also observed for some subjects. These large errors would arise because inappropriate pulse waveforms are obtained for some reasons. If the U-shaped box is not placed in the right position, for example, one leg of it touching the silicone sheet above the sensing FBG, the pulse waveform would be distorted. On the other hand, if the clamp presses the U-shaped box and, accordingly, the wrist is too tight, the BPs would be measured incorrectly because of artery contraction. We carefully operated the test by gradually increasing the clamping force until clear pulse waveforms were obtained, thus avoiding pressing the wrist too hard. Also, the position of the sensing FBG in the silicone sheet was marked in the test to avoid misplacement of the U-shaped box. We believe that the slight error of 5 mmHg in the BP measurement with PTT, Pt, and Dt all considered in the estimation model for the 29 subjects was caused by the difference between the BPs acquired from the two hands, especially when the BPs were taken by inflation and deflation of a cuff around the right arm, in which case the blood vessel could be closed temporarily and this affect the BP measurement on the left hand.

## Figures and Tables

**Table 1:** Ptt, Pt, and Dt derived from pulse waveforms of 29 subjects, as well as their heart rates derived from their pulse waveforms and those obtained by Omron.

Subject	PTT (s)	Pt (s)	Dt (s)	Heart Rate Derived from Pulses (s^−1^)	Heart Rate Obtained by OMRON (s^−1^)
A	0.272	0.075	0.285	89	85
B	0.272	0.090	0.295	89	84
C	0.263	0.090	0.300	93	89
D	0.249	0.090	0.540	85	82
E	0.276	0.080	0.415	73	70
F	0.274	0.095	0.315	91	87
G	0.253	0.120	0.455	93	90
H	0.238	0.085	0.370	85	83
I	0.255	0.125	0.310	93	91
J	0.263	0.075	0.355	84	86
K	0.274	0.085	0.520	70	70
L	0.245	0.090	0.350	85	84
M	0.256	0.095	0.305	84	81
N	0.261	0.095	0.365	82	80
O	0.262	0.135	0.280	86	85
P	0.262	0.085	0.345	84	84
Q	0.280	0.130	0.320	78	77
R	0.256	0.090	0.355	87	84
S	0.288	0.100	0.335	85	82
T	0.224	0.095	0.185	119	115
U	0.217	0.125	0.360	82	85
V	0.288	0.095	0.190	X	X
W	0.256	0.115	0.305	X	X
X	0.235	0.085	0.225	X	X
Y	0.260	0.090	0.455	X	X
Z	0.250	0.085	0.375	X	X
Z1	0.239	0.095	0.455	X	X
Z2	0.231	0.075	0.390	X	X
Z3	0.245	0.125	0.385	X	X

## Data Availability

The data presented in this study are available in this article.
